# Release of HSV-1 Cell-Free Virions: Mechanisms, Regulation, and Likely Role in Human-Human Transmission

**DOI:** 10.3390/v13122395

**Published:** 2021-11-30

**Authors:** Stephen A. Rice

**Affiliations:** Department of Microbiology and Immunology, University of Minnesota, Minneapolis, MN 55455, USA; ricex019@umn.edu

**Keywords:** HSV-1, HSV-2, cell-free virions, cell-cell spread, glycoproteins, heparan sulfate, heparanase, human-human transmission, varicella-zoster virus, Marek’s disease virus

## Abstract

Herpes simplex virus type 1, or HSV-1, is a widespread human pathogen that replicates in epithelial cells of the body surface and then establishes latent infection in peripheral neurons. When HSV-1 replicates, viral progeny must be efficiently released to spread infection to new target cells. Viral spread occurs via two major routes. In cell-cell spread, progeny virions are delivered directly to cellular junctions, where they infect adjacent cells. In cell-free release, progeny virions are released into the extracellular milieu, potentially allowing the infection of distant cells. Cell-cell spread of HSV-1 has been well studied and is known to be important for in vivo infection and pathogenesis. In contrast, HSV-1 cell-free release has received less attention, and its significance to viral biology is unclear. Here, I review the mechanisms and regulation of HSV-1 cell-free virion release. Based on knowledge accrued in other herpesviral systems, I argue that HSV-1 cell-free release is likely to be tightly regulated in vivo. Specifically, I hypothesize that this process is generally suppressed as the virus replicates within the body, but activated to high levels at sites of viral reactivation, such as the oral mucosa and skin, in order to promote efficient transmission of HSV-1 to new human hosts.

## 1. Herpes Simplex Virus Type-1, an Important Human Pathogen

Herpes simplex virus type-1 (HSV-1) is a widespread human alphaherpesvirus that infects the majority of the U.S. population by adulthood [1]. In most individuals, HSV-1 infection results in mild disease, such as herpes labialis, the vesicular lesions on or near the lips that are commonly known as cold sores. However, the virus is also capable of causing much more serious illnesses, including herpes stromal keratitis, an eye disease resulting in 500,000 U.S. physician visits/year [2]; herpes encephalitis, an infection of the brain lining that is often fatal if not treated promptly [3,4]; and disseminated neonatal infections, which can be devastating to newborns [5]. Additionally, HSV-1 is increasingly investigated as an etiological factor in Alzheimer’s Disease [4,5,6]. Moreover, a close cousin of HSV-1, HSV-2 is a serious human pathogen as well as a risk factor for acquiring human immunodeficiency virus-1 (HIV-1) infection [7,8].

Although there are effective antivirals against HSV-1 and -2, such as acyclovir [9], there is a need to develop additional therapeutic approaches. This is because the current anti-HSV drugs are unable to eradicate established infections and are subject to evasion by viral resistance mutations [9,10]. Furthermore, there are presently no licensed vaccines for HSV-1 or -2. This review will focus on an area of HSV-1 biology that has not been heavily studied: the release of viral progeny from infected cells as cell-free particles. It is feasible that an increased understanding of this process could present new opportunities for antiviral therapy.

The broad outlines of HSV-1’s interaction with its human host are well known [11,12]. Typically, primary infection occurs during childhood and involves the transfer of infectious bodily fluids (usually saliva) from an infected individual to a naïve one. The physical agents of transmission are believed to be cell-free virions [11,12,13]. Infection is most often established in epithelial cells of the skin or mucosae, often in the oral region [14], and may be enhanced by tissue damage. After a period of replication in the epithelium, which is often mild or asymptomatic, the virus infects neurons of the peripheral nervous system (PNS) that interface with the epithelium, entering these cells at their termini. Once inside neurons, viral nucleocapsids traffic on microtubules to neuronal cell bodies. Viral genomes then enter neuronal nuclei, where they establish a lifelong latent infection. During latency, the HSV-1 chromosome is maintained as a covalently closed episome that undergoes only limited gene expression [15]. However, at sporadic intervals during the lifetime of the host, often associated with stress, latent HSV-1 can undergo reactivation, a process wherein the virus reinitiates productive replication and produces virions that are released from neuronal termini to re-seed infection at the epithelium, usually at or near the site of primary infection [16]. Cell-free virions shed from the epithelium during reactivation are the agents responsible for human-human transmission. The interaction of HSV-2 with humans is quite similar to that of HSV-1, although for this virus the genital tract is the usual site of infection [8].

## 2. Structure and Cell Entry of HSV-1 Virions

**Figure 1:**
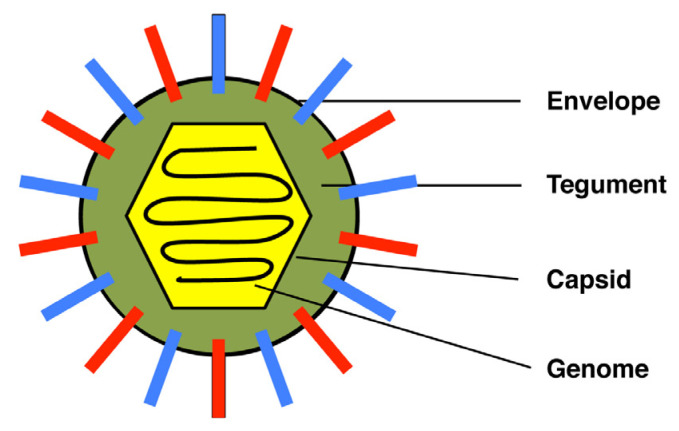
Structure of the HSV-1 virion. Envelope proteins are represented by red and blue bars. See text for further details.

HSV-1 virions are quasi-spherical particles ~200 nm in diameter, consisting of four distinct layers (Figure 1). The outermost layer is the envelope, a lipid bilayer derived from the host cell. The envelope has 17 virus-encoded proteins embedded in it, 12 of which are glycoproteins [17,18]. The envelope proteins have an organized architecture on the surface of the virion that is not yet fully characterized [19,20]. Beneath the envelope is the tegument, a complex proteinaceous layer comprised of >20 viral polypeptides and even some host proteins [17,21]. Tegument proteins are crucial for virion assembly and many also carry out important replicative functions. For example, some tegument proteins engage cellular microtubular motor proteins to allow nuclecapsids to traffic through the cytoplasm [17]. Other tegument proteins are released into the cytoplasm early in infection and regulate host and viral gene expression. Beneath the tegument is the capsid, an icosahedral shell comprised of eight viral proteins [22]. Inside this is the fourth and innermost layer, the 152 kb linear double-stranded viral genome, which encodes ~85 viral proteins.

To understand the release and spread of HSV-1, a discussion of how released progeny virions enter new target cells is necessary. Cell entry by HSV-1 is a highly regulated process involving several viral and host molecules (reviewed in [18,23,24,25]). It can be divided into two steps: (i) cell attachment and (ii) membrane penetration. In cell attachment, HSV-1 virions bind to heparan sulfate (HS) moieties on cell surface polypeptides [24,26]. Such sugar-protein complexes are termed HS-proteoglycans (HSPG) and are nearly ubiquitous on the surface of mammalian cells [27]. To bind to HSPG, virions use envelope glycoproteins C (gC) and/or gB, both of which interact electrostatically with HS via positively-charged regions on their surfaces [24,27,28,29,30,31]. This relatively non-specific interaction serves to concentrate virions on the cell surface and enhances HSV-1 entry but is not strictly required for infection [32]. However, the second entry step, membrane penetration, is essential. In this step, the viral envelope fuses with the plasma or endosomal membrane of the cell, depositing the tegument-coated nucleocapsid into the cell cytoplasm. Four viral envelope glycoproteins—gB, gD, gH, and gL—are necessary for membrane penetration and hence can be said to comprise the core entry machinery of HSV-1 [23,33]. The critical membrane fusion step is catalyzed by gB, which is a member of the class III fusion protein family [34]. However, gB’s fusogenic activity is carefully regulated such that viral entry occurs only under appropriate conditions. To initiate the process, gD must first bind to one of three host cell entry receptors: nectin-1, herpesvirus entry mediator (HVEM), or 3-O-sulfated HS [23]. This interaction then signals to a heterodimer composed of gH and gL, which functions as a crucial gB regulator. The gH-gL dimer then physically interacts with gB in a manner that drives its fusogenic activity, allowing membrane fusion and the entry of HSV-1 into the target cell.

## 3. Two Major Modes of HSV-1 Spread: Cell-Free Release and Cell-Cell Spread

Enveloped animal viruses exhibit two primary modes of transmission between cells, cell-free release (CFR) and cell-cell spread (CCS), as well as some other modes of viral spread that are less characterized [35,36,37]. In CFR, progeny virions are released into the extracellular environment and can travel by diffusion or other means to infect new target cells. In CCS, progeny virions are delivered directly to cell-cell junctions (e.g., tight junctions, adherens junctions, desmosomes) for spread to adjacent cells. CCS can occur in highly polarized cells (e.g., polarized epithelia, neurons) as well as in non-polarized cells (e.g., lymphocytes). It can be advantageous as a means of spread, because it usually occurs in a spatial context that is shielded from the extracellular milieu, thus protecting virions from neutralizing antibodies and other soluble immune effectors. CCS also minimizes the need for progeny virions to be constructed to be stable in the extracellular environment. Furthermore, the highly directed nature of CCS likely increases the multiplicity of infection in the downstream target cell, thereby increasing the likelihood of productive infection [38].

**Figure 2:**
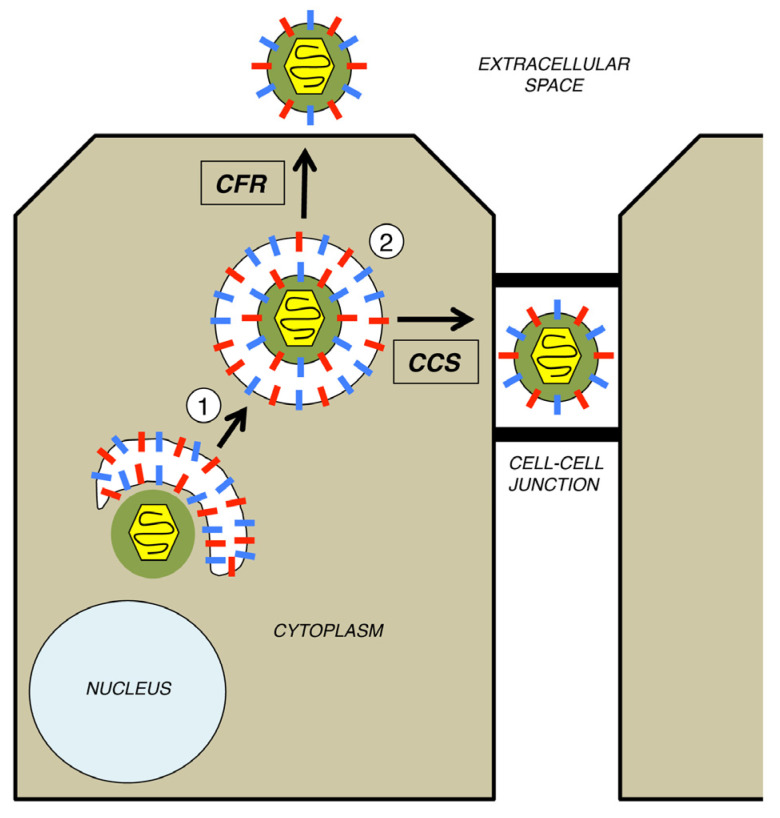
Two major modes of HSV-1 spread. For simplicity, only the late cytoplasmic stages of HSV-1 assembly and egress are shown. In step 1, the cytoplasmically localized, tegument-coated nucleocapsid undergoes secondary envelopment at the membrane of a cytoplasmic organelle, resulting in a virion within a vesicle. In step 2, the vesicle traffics either to the cell surface (for CFR) or to a cell-cell junction (for CCS), for release by exocytosis.

HSV-1 uses both CFR and CCS [39]. To understand the pathways involved, one must appreciate that virion release and spread are the final events in the complex process of HSV-1 assembly and egress that begins in the infected cell nucleus. There, viral DNA is replicated and packaged into preformed capsids. These nucleocapsids then traverse the bilamellar nuclear membrane using a pathway of envelopment and de-envelopment [40]. It is not until nucleocapsids arrive naked in the cytoplasm that the events relevant to this review take place. CFR and CCS share two mechanistic steps (Figure 2) [39,41,42]. The first is secondary envelopment, a process wherein the nucleocapsid, now associated with tegument proteins, buds into the membrane of a cytoplasmic organelle, generating an enveloped virion inside a vesicle (Figure 2, step 1). Key to driving this process are the physical interactions between the viral tegument proteins and the cytoplasmic domains of the viral envelope proteins, which, following their biosynthesis, are embedded into the membrane of the enwrapping organelle. The identity of the membranous organelle at which secondary envelopment occurs has been the subject of debate [17,21,41]. One long-standing model is that envelopment occurs at vesicles derived from the trans-Golgi network [43]. A more recent proposal is that it occurs at membrane tubules originating from recycled endosomes [44,45]. In either case, the membranes used for secondary envelopment must contain all of the viral envelope proteins that are found on the mature virion. After secondary envelopment, the virion becomes infectious, but it is trapped within a vesicle inside the cell. Thus, in a subsequent step (Figure 2, step 2), the vesicle traffics to the cell periphery, where it is released by exocytosis, the cellular process wherein a vesicle’s membrane fuses with the plasma membrane of the cell. This occurs at either a cell-cell junction for CCS (rightward arrow) or the cell surface for CFR (upward arrow).

Similar to many other enveloped viruses, HSV-1 makes heavy use of CCS [39]. This mode of spread has been documented for HSV-1 in epithelial cells [46] and neurons [47], as well as between neurons and epithelial cells [48]. Moreover, CCS is important for HSV-1 pathogenesis, as HSV-1 mutants defective in CCS produce abnormally small lesions in the cornea of infected rabbits and mice [46]. Several viral proteins are required for efficient CCS, including a heterodimer of two glycoproteins, gE and gI [46], and tegument proteins pUL51 and pUL7 [49]. Although the exact mechanism of HSV-1 CCS is not known, the prevailing models [49,50,51] suggest that the viral envelope and tegument proteins mentioned above help direct virion-containing vesicles to cell-cell junctions, where they are released into small, sterically protected intracellular spaces, where virion interactions with host entry receptors can occur efficiently. This is consistent with studies showing that CCS requires the HSV-1 core entry machinery (i.e., gB, gD, gH, and gL) [52].

## 4. Mechanisms and Regulation of HSV-1 Cell-Free Virion Release

**Figure 3:**
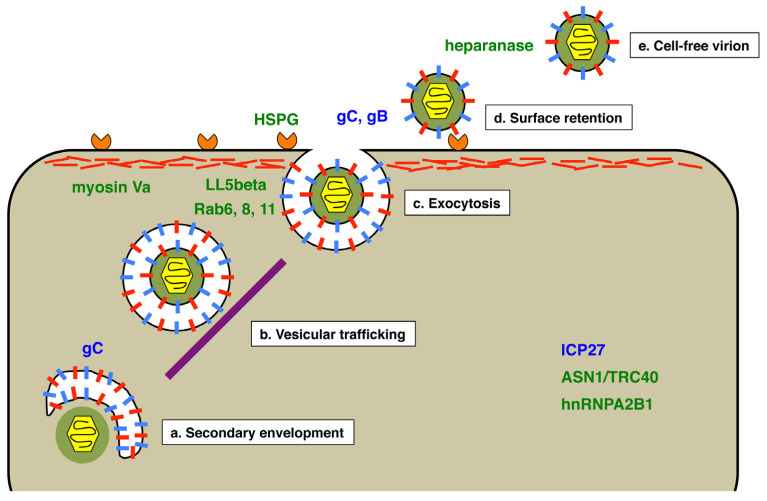
Proposed pathway of HSV-1 cell-free virion release. The schematic diagram shows the cytoplasm of an infected cell, illustrating five steps (**a**–**e**) in the production of HSV-1 cell-free virions, beginning with secondary envelopment of the tegumented-coated capsid. The purple bar represents microtubules involved in trafficking vesicles to the plasma membrane, whereas the network of red lines at the top of the cell represents the actin cortex. Factors implicated in mediating or regulating cell-free virion release are indicated in blue for HSV-1 factors and in green for host factors.

Some of the earliest cell culture studies of HSV-1 in the 1950s revealed that a significant fraction of viral progeny is released into the media as cell-free virus [53]. Since then, similar observations have been made in scores of other in vitro studies. However, it is doubtful whether robust CFR is a general feature of HSV-1 infection in vivo, because in most individuals, viral infection is well confined to surface epithelia and neurons. Supporting this view, it was found that many newly isolated clinical strains of HSV-1 are deficient in CFR, spreading primarily by CCS when first placed in cell culture [54]. These observations suggest that CFR should not be seen as the default mechanism for HSV-1 release. Rather, it is better viewed as a specialized pathway for viral release and spread, similar to CCS, which is likely subject to regulation by the virus. In this section, I review current knowledge of the pathway of HSV-1 CFR (Figure 3), with discussion of possible points where regulation of the process might be exerted.

As discussed earlier, CFR depends on a secondary envelopment event within the infected cell cytoplasm, leading to an HSV-1 virion within a vesicle (Figure 3a). The vesicle must then engage a kinesin motor to travel on microtubules to the plasma membrane (Figure 3b). How these events differ for virions destined for the cell surface versus those bound for cell-cell junctions is not known. However, from studies of CCS, it is clear that the composition of the nascent virion, in terms of its envelope and tegument proteins, can determine the destination of delivery. For example, gE and gI promote the delivery of virions to cell-cell junctions [46,55]. It is thus conceivable that one or more envelopes or tegument proteins enhance delivery of virions to the plasma membrane for CFR. One candidate is envelope protein gC. This is supported by studies of related herpesviruses that suggest their gC homologs promote CFR (discussed further in the following section) [56,57]. Additionally, it is worth noting that HSV-1 gC deletion mutants form larger plaques than the wild-type virus, suggesting that they have enhanced CCS [31]. This could be explained if there is a competition between CFR and CCS at the stage of secondary envelopment and/or vesicular trafficking. Such a competition is supported by the finding that a CCS-deficient gE mutant exhibits unusually high levels of CFR [49].

Ultimately, vesicles containing HSV-1 virions arrive at the plasma membrane and release their contents into the extracellular environment by exocytosis (Figure 3c). This process was investigated by Hogue et al. in live-cell imaging studies of pseudorabies virus (PRV), a swine alphaherpesvirus related to HSV-1 [58,59]. The authors used a PRV strain that encodes both a GFP-tagged envelope protein and an RFP-tagged capsid protein, allowing them to visualize virions in live cells. Since the fluorescence of the envelope-tag was dependent on neutral pH, it was possible to observe the precise moment of exocytosis, when an infectious virion emerged from the acidic vesicle and entered the pH-neutral extracellular environment. The work led to three significant findings relevant to the exocytosis of alphaherpesviruses. First, virion-transport vesicles exhibit rapid, directional transport to the plasma membrane. Second, nearly all exocytic events involve vesicles carrying only a single virion. The third finding relates to the cellular Rab GTPases, which are involved in virion release. This large family of membrane-bound proteins are critical regulators of membrane trafficking and are found on various membrane compartments [60]. Using a co-localization approach, the authors implicated Rab6a, Rab8a, and Rab11 in the trafficking of virions to the plasma membrane. All three are known to be involved in constitutive secretory trafficking in uninfected cells, suggesting that alphaherpesviruses reach the cell surface using a normal host pathway. The use of a constitutive host mechanism may help explain how these viruses breach the actin cortex, the cytoskeletal structure underlying the plasma membrane that presents a theoretical impediment to viral exit. It should be mentioned that a prior study provided evidence that myosin Va also plays a role in allowing HSV-1 to pass through the actin cortex [61].

Where on the plasma membrane are HSV-1 virions released? Although the answer is unknown, several studies suggest that the sites of virion exocytosis are not random and may occur preferentially at regions associated with the cytoskeleton. In one study performed in non-polarized Vero epithelial cells, released HSV-1 virions accumulated at specific pocket-like areas of the plasma membrane that were associated with either the physical substratum or with adjacent cells [62]. In their PRV studies, Hogue et al. also found evidence for release at specific sites, in this case near patches of LL5beta, a host protein known to help anchor microtubules to the plasma membrane [58].

In theory, the exocytosis of HSV-1 virions should complete their release into the soluble environment. However, multiple studies show that released virions remain tightly bound to the outside surface of the cell for a prolonged period [58,63,64,65]. Thus, surface retention should be viewed as a normal step in the pathway of HSV-1 CFR (Figure 3d). Supporting this, Park et al. found that, at times well after HSV-1 infection of Vero cells, most of the total viral progeny (>80%) was attached to the external cell surface, with nearly all of the remainder being released into the culture supernatant [65]. Surprisingly, almost no infectious virus (<0.5%) could be detected inside the infected cell. This study suggests that the infected cell surface is a major repository for the infectious viruses produced in an HSV-1 infection.

What are the molecular interactions that retain progeny virions on the cell surface? The most obvious possibility is that virions are tethered by the same interaction that serves to attach them at the beginning of infection, i.e., their binding to HSPG via envelope proteins gC and/or gB. Supporting this idea, it was shown that a significant amount of viral progeny can be released from HSV-2-infected cells by washing the cells with buffers containing heparin, an HS mimic [66]. An intriguing study from the Shukla group also supports HSPG-based retention of virions and further provides a mechanism to explain how such retention can be overcome to allow virions to be released into the extracellular milieu [67]. The authors found that HSV-1 infection up-regulates the expression of cellular heparanase, the only enzyme known to be capable of cleaving HS from cells. Moreover, infection was shown to induce the relocalization of heparanase from inside the cell to the cell surface. Importantly, these events correlated with the release of HSV-1 into the media, strongly suggesting that heparanase directly severs the linkage between progeny virus and host cell. Similar findings were made by the same group for HSV-2 [68]. Thus, heparanase appears to solve a problem for HSV-1/2 that is theoretically encountered by all viruses [69]. That is, given that viruses bind to specific ligands on the cell surface to begin infection, how do they avoid being entrapped by the same interactions as they exit the cell at the end of infection? The best-characterized solution to this problem is provided by influenza A virus (IAV), which encodes an envelope-bound neuraminidase [70]. It is thought that as a progeny IAV virion exits the cell, its neuraminidase component cleaves sialic acid off the cell surface. Since sialic acid is the IAV receptor, this clears the way for efficient virion release. The studies of Shukla and colleagues strongly suggest that heparanase performs a similar role for HSV-1/2.

After their detachment from the infected cell, HSV-1 progeny virions can be accurately described as “cell-free”. (Figure 3e). However, work by Newcomb and Brown showed that HSV-1 virions can undergo post-release maturation events [71]. These authors used both electron microscopy and biochemical analysis to show that cell-associated and cell-free virions are distinctly different in their physical properties. Cell-associated virions were found to possess a tegument that is uniformly distributed around the capsid, whereas that of cell-free virions was shown to be markedly asymmetric. Moreover, the tegument of cell-free virions was resistant to detergent extraction compared to that of cell-associated virions. Although the significance of theses differences is unknown, it was suggested that they reflect a viral mechanism to enhance infectivity in the harsh external environment [71]. Supporting this idea, another study by the same group found that a small amount of enzymatically active host catalase is packaged into the HSV-1 tegument [72]. This enzyme can detoxify hydrogen peroxide and thus might serve to protect extracellular virions from oxygen-induced damage.

To round out the discussion of HSV-1 CFR mechanisms, it should be mentioned that there are some viral and cellular factors that have been shown to affect CFR, but for which little is known about the mechanisms involved. One viral factor is immediate-early protein ICP27. The evidence for this comes from the study of an HSV-1 strain engineered to encode the HSV-2 ICP27 in place of the normal protein [65]. Surprisingly, the only phenotype seen for this mutant was a deficiency in CFR, leading to an altered plaque morphology. Two host proteins have been shown to promote CFR. One is ASNA1/TRC40, a factor required for the membrane-insertion of tail-anchored membrane proteins [73]. When ASN1/TRC40 expression was reduced by RNAi, HSV-1 replication occurred normally, but there was a dramatic defect in CFR. Similarly, knockout of the gene for hnRNPA1B2, a cellular RNA-binding protein, led to a significant CFR deficit upon HSV-1 infection [74]. More work is required to determine how these cell factors and ICP27 modulate CFR.

## 5. Can HSV-1 Regulate Its Mode of Spread In Vivo? Clues from Other Herpesviruses

HSV-1 can spread by either CFR or CCS, but it is not known whether it can modulate its mode of release within an infected host. However, work on some other herpesviruses suggests that the answer is likely yes. Some of the most intriguing work comes from the study of human cytomegalovirus (HCMV), a member of the betaherpesvirus family. This virus is widespread in the human population and capable of causing significant morbidity and mortality in immunocompromised hosts [75,76]. HCMV exhibits wide tissue tropism in vivo, which is at least partially explained by its ability to enter many different types of cells. The HCMV entry machinery is evolutionarily related to that of HSV-1, in that the virus encodes homologs of gB, gH, and gL [23]. However, unlike HSV-1, which uses a single entry mechanism, HCMV has evolved two distinct entry mechanisms, and each utilizes a different viral envelope protein complex [23,75,76]. One complex is known as the “trimer” and consists of the gH-gL heterodimer bound to an HCMV-specific glycoprotein, gO. The other is termed the “pentamer” and consists of gH-gL bound to HCMV-specific proteins UL123/130/131. Importantly, each complex binds to a different cellular receptor and mediates entry into different cell types. After receptor engagement, both complexes interact with gB to trigger membrane fusion. Of particular interest in this discussion is that the infectivity of HCMV cell-free virions is strongly dependent on the presence of the trimer complex [77,78]. Thus, gO-negative viral mutants are viable but unable to spread by the cell-free route. Interestingly, there is evidence that, in many cell types, gO levels are constitutively kept low due to the inherent susceptibility of gO to proteolysis by the cell’s endoplasmic reticulum-associated degradation (ERAD) machinery [79]. Consistent with this, the spread of HCMV within infected humans is primarily by CCS [76]. However, HCMV encodes a protein, UL148, that interacts with the ERAD machinery in a manner that stabilizes gO [79]. Thus, gO accumulates in the presence of UL148, resulting in infectious cell-free virions. Based on these findings, it was hypothesized that UL148 is important for the HCMV horizontal spread [76,79]. For example, if UL148 is expressed in the epithelia of secretory tissues, it could activate the infectivity of cell-free virions in the bodily fluids (saliva, urine, and breastmilk) known to be responsible for transmission of HCMV to new hosts.

Additional evidence for modulation of viral spread in the infected host comes from the study of varicella-zoster virus (VSV), another human alphaherpesviral pathogen. VZV is responsible for causing both varicella (chickenpox) and zoster (shingles) [80,81]. The primary infection, which is associated with chickenpox, begins in epithelial cells of the upper respiratory tract. Soon, the virus is seeded to T cells, which typically traffic infection to the skin. There, virions gain access to sensory neurons, where they establish lifelong latency. Reactivation of the virus later in life can result in zoster, a painful vesicular skin rash. In cell culture, VZV is notoriously cell-associated, with little release of cell-free virions [82]. This is the case even in human infections in vivo, with one significant exception: vesicular skin lesions exhibit high concentrations of cell-free virions [83,84]. These can be transmitted to new hosts via aerosolization (unlike HSV-1, which requires direct human-human contact). It is not understood why VZV CFR is highly restricted in most cells, but it has been shown that virion egress is often inefficient, with many virions being degraded in late endosomes [83]. Interestingly, the replication of VZV in human skin cells requires the VZV gC homolog [56,85], as does efficient CFR [56]. It is noteworthy that in many cultured cells the VZV gC gene is non-essential for replication and has an idiosyncratic expression pattern that is characterized by highly delayed and low expression relative to most other VZV genes [86,87,88]. In contrast, in human skin lesions, gC is expressed robustly [87]. Together, these results suggest VZV gC is up-regulated in human skin, where it plays a key role in activating CFR in order to promote host-host transmission.

Perhaps the most definitive evidence that herpesviruses can regulate their mode of spread in vivo comes from work on Marek’s Disease Virus (MDV), an avian alphaherpesvirus. The MDV system is particularly valuable because it provides a robust host-host transmission model [57], something not readily achievable with human herpesviruses. In chickens, MDV is a notorious pathogen that causes a variety of symptoms, including wasting, neurological defects, and T cell lymphomas [57]. Infection begins in the respiratory tract when chickens inhale dander emanating from infected chickens. After a complex replication pathway that includes latency in T cells, MDV infection is established in the feather follicle epithelial (FFE) cells of the skin, which are the source of the shed dander that is responsible for horizontal transmission. Although MDV is highly cell-associated in cultured cells and in most tissues of the chicken, cell-free virions are produced in FFE cells [89], likely explaining why this tissue is key to horizontal transmission [90]. Intriguingly, even though MDV gC is not required for either replication or disease in the chicken, it is essential for horizontal spread [90,91,92]. This suggests that its role is to enhance the production of infectious cell-free virions in FFE cells [90,93]. The MDV gC gene is similar to the VZV gC gene in that it is expressed poorly in cell culture [57]. It is also subject to deletion and silencing as the virus is passaged in the laboratory [57]. Two other MDV proteins, the ICP27 homolog and pUL47, a tegument protein, are also needed for efficient horizontal transmission of MDV in chickens [94,95]. Interestingly, both regulate expression of the MDV gC mRNA. Overall, the studies of MDV indicate that this virus has evolved a mechanism to activate CFR in a specific cell type, FFE cells, to promote horizontal transmission. Moreover, the MDV gC protein plays an essential but as yet undefined role in this process.

## 6. Hypothesis: Release of Cell-Free HSV-1 Is Induced at Sites of Viral Reactivation

The high prevalence of HSV-1 in humans indicates that it is a master at transmitting itself to new hosts. In part, this can be attributed to the life-long nature of the infection. However, it also suggests that HSV-1 has evolved an efficient mechanism to shed progeny virions in the tissues associated with transmission. Although HSV-1 shedding has not been well studied, clues to this process can be gleaned from work on HSV-2 shedding that has been carried for more than 20 years by investigators at the University of Washington (UW) [8,13,96,97,98,99,100,101]. A groundbreaking initial study enlisted HSV-2-positive individuals to swab their genitalia over the course of many weeks and provide the samples to the clinic for PCR-detection of virus [101]. The results, which were quite surprising at the time, demonstrated that HSV-2 genital reactivation events are much more frequent than previously thought and are often asymptomatic.

Since then, the HSV-2 shedding studies at UW have advanced in several ways and continue to lead to new insights. First, sampling protocols were broadened to increase the frequency of testing as well as the spatial detail of the anatomical surfaces assayed (e.g., in some studies, individuals swabbed >20 distinct but contiguous areas of their epithelia) [97,99]. A key insight to emerge from this work was that HSV-2 reactivation is extremely heterogenous, even in a single individual, in regards to the location, strength, and duration of the events. A second advance was the use of biopsies to identify host immune cells that respond to HSV-2 reactivation in the epithelium [97,100,102,103]. From this work, researchers identified HSV-2-specific tissue resident memory CD8+ T cells (TRMs) as key players in controlling viral reactivation [13,100]. TRMs are a subset of CD8+ T cells that are permanently localized to specific tissues and serve as first responders to fight off reinfections at body surfaces [104]. It was found that in nearly all reactivation events, TRMs result in contraction of viral loads within 12–24 h. However, TRMs are not evenly dispersed in the tissue, but rather clustered in heterodispersed aggregates at the dermal-epidermal boundary, and in some cases are not well positioned to quickly contain HSV-2 emergence [105]. This heterogeneity gives the virus a small window of opportunity to evade immune control, at least temporarily.

A third advance in the HSV-2 shedding studies was the development of mathematical models of genital reactivation [97,105,106]. These have been reiteratively designed to fit the clinical data and can be used to identify key questions and generate testable hypotheses. The most advanced model, described by Schiffer et al. [13], begins to provide a coherent picture of the complex dynamics that accompany an HSV-2 reactivation event in the genital epithelium. It assumes that latently infected neurons in the dorsal root ganglion provide a steady drip of reactivating HSV-2 into the genital epithelium [106]. The heterogeneity of the resulting epithelial reactivation events, in terms of strength and duration, is primarily driven by time and spatial constraints on TRMs to contain each reactivation. Here, the mode of spread becomes important. The model assumes that HSV-2 can spread using either CCS or CFR. However, CFR is hypothesized to be crucial to temporarily outrunning immunity, as cell-free virions can seed adjacent regions of the epithelium that have lower TRM density, leading to the formation of secondary lesions and further spread [97]. TRMs ultimately control all reactivation events, but not before the occasional production of sufficient progeny to drive transmission to new hosts. Referring to the battle between HSV-2 and the host in the human genital epithelium, Schiffer et al. state that, although the “immune system ultimately wins each battle”, the “virus wins the war”, because it so adept at horizontal transmission [13].

Given its high similarity to HSV-2, it is likely that HSV-1 uses a similar strategy to outrun host TRMs during epithelial reactivation events. I thus hypothesize that HSV-1 has evolved a mechanism to strongly induce CFR at sites of viral reactivation, such as the oral mucosa and skin. This could involve an increase in the production of cell-free virions (similar to what occurs for VZV in human skin lesions), or it could reflect an increase in the infectivity of cell-free virions (similar to what is hypothesized for HCMV). It is likely that cellular context plays an important role in the mechanism. For example, activation of CFR may depend on specific host factors expressed in keratinocytes or mucosal epithelial cells. Supporting this idea, ex vivo infection of skin explants by HSV-1 was found to result in high CFR, and this mode of release appeared to be required for spread in this tissue [107]. In contrast, CFR was not seen in HSV-1-infected brain tissue, where spread occurred exclusively by CCS.

**Figure 4:**
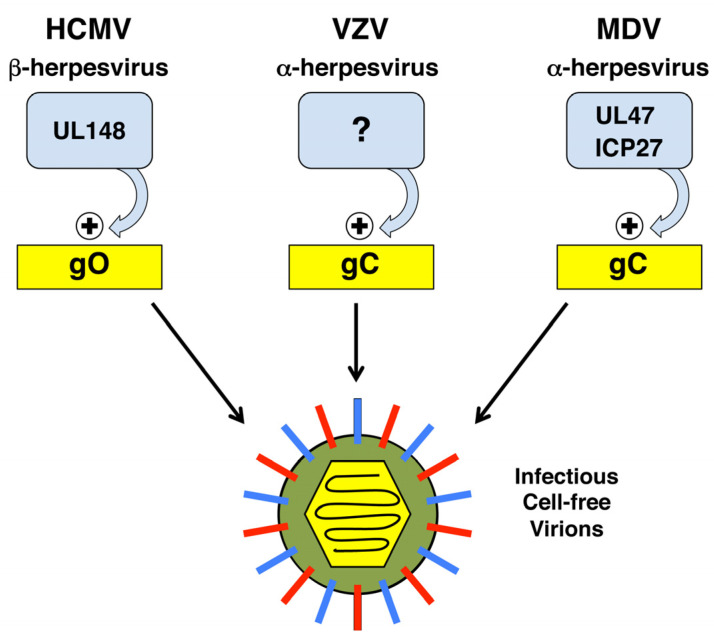
A common theme in the regulation of CFR by diverse herpesviruses. See text for details.

How might HSV-1 induce CFR? Important clues come from the studies of HCMV, VZV, and MDV. Comparison of these systems reveals a common theme for how diverse herpesviruses can activate CFR, with several key features (Figure 4). First, in all three cases, there is evidence that CFR depends on the presence of a non-essential envelope glycoprotein (gO for HCMV; gC for VZV and MDV). Second, the glycoproteins in question all appear to be under strong regulatory control, in some cases leading to a near on/off state in their expression (ERAD regulation of gO protein levels; transcriptional/post-transcriptional regulation of VZV/MDV gC mRNA). Third, in the case of HCMV and MDV, there is evidence that specific trans-acting viral proteins positively regulate expression of the glycoproteins (UL148 for gO, UL47 and ICP27 for MDV gC).

Given these clues, it is intriguing to speculate that, like its alphaherpesviral relatives, HSV-1 utilizes gC to promote CFR. In this regard, it is noteworthy that the HSV-1 gC gene is subject to strong transcriptional and post-transcriptional regulation, in some cases mediated by ICP27 [108,109,110,111,112]. Given that ICP27 modulates HSV-1 CFR through an as yet unknown mechanism [65], it is worth investigating whether ICP27-gC might comprise a regulatory axis to induce CFR in the tissues associated with reactivation.

## 7. Concluding Remarks

In this article, I have attempted to synthesize the current knowledge of the mechanisms of HSV-1 CFR. This is an important topic, as cell-free virions are almost certainly the agents of HSV-1 human-human transmission. It is possible that therapeutics could be developed to target CFR as a means to both alleviate HSV-1 disease and suppress horizontal spread. Possible approaches could be to directly target cell-free virions with virucidal agents [113,114] or to inhibit elements of the CFR pathway. With regard to the latter possibility, it is worth noting that host heparanase expression has been linked to metastatic cancer [115], prompting the fairly advanced development of several heparanase inhibitors [116]. It would be interesting to see if any of these agents inhibit HSV-1 or -2 replication, spread, or pathogenesis in animal models.

Although CFR is likely key for HSV-1 human-human transmission, it is poorly suited as a mode of spread within the human body. For this reason, it is likely that CFR is suppressed in vivo to ensure that the infected host survives in a healthy state to spread infection to new hosts via normal interactions with other humans. Indeed, uncontrolled spread of HSV-1 and -2, in the form of disseminated infections, is associated with severe disease and death, particularly in neonates [5]. It is possible that this disease state is associated with a disruption of the normal circuits that control HSV-1 spread. It is hoped that this review will spur future research into how HSV-1 spread is regulated, a fascinating but under-studied aspect of the biology of this important human pathogen.
